# Transformation of phytochemicals and functional properties in *Eleutherococcus senticosus* leaves during solid-state fermentation with *Rhizopus oligosporus*

**DOI:** 10.1007/s10068-026-02276-9

**Published:** 2026-08-13

**Authors:** Suprakash De, Hayeong Kim, Ghahyun Jeffrey Kim, Taeyoung Um, Yedam Moon, Kibeom Jin, Sooye An, Kunal Pal, Doman Kim

**Affiliations:** 1 Graduate School of International Agricultural Technology, Seoul National University, Gangwon-Do, 25354 Republic of Korea; 2 Institutes of Green Bioscience and Technology, Seoul National University, Gangwon-Do, 25354 Republic of Korea; 3 Department of Clinical Pharmacy Practice, School of Pharmacy &Pharmaceutical Sciences, UC Irvine, Irvine, CA 92617 USA; 4 Department of Plant Science, Kangwon National University, Gangneung, 25457 Republic of Korea; 5 Agricultural Corporation Soye Pyeongchang Ogapi Co. Ltd, Pyeongchang, 25354 Republic of Korea; 6 Falculty Department of Biotechnology and Medical Engineering, National Institute of Technology Rourkela, Rourkela, 769008 Odisha India; 7 Fervere Campus Corporation, Gangwon-Do, 25354 Republic of Korea

**Keywords:** Antioxidant, *Eleutherococcus senticosus* leaf, L-carnitine, *Rhizopus oligosporus*, β-sitosterol

## Abstract

*Eleutherococcus senticosus* (*E. senticosus*) leaves were subjected to solid-state fermentation with *Rhizopus oligosporus* KCCM11948P for 120 h to evaluate changes in phytochemicals and functional properties. Fermentation increased water-swelling, water-holding, cholesterol-binding, and oil absorption capacities. Chlorogenic acid decreased by 33.7%, whereas caffeic acid increased by 33.5% after 72 h of fermentation. L-carnitine was first detected at 72 h and reached 15.67 ± 0.50 mg/kg DM after 120 h, while β-sitosterol increased by 105.2%. Total phenolic and flavonoid contents increased by 38.6% and 20.8%, respectively. Antioxidant activities measured by the FRAP, ABTS, and ORAC assays increased throughout fermentation, and nitric oxide (NO)-inhibitory activity in RAW264.7 macrophages was enhanced by 83.0% compared with non-fermented leaves. These results demonstrate that solid-state fermentation with *R. oligosporus* improves the phytochemical composition and functional properties of *E. senticosus* leaves, supporting their potential use as functional ingredients.

**Supplementary Information:**

The online version contains supplementary material available at https://doi.org/10.1007/s10068-026-02276-9.

## Introduction

*Eleutherococcus senticosus* (*E. senticosus*), commonly known as Siberian ginseng, is a widely distributed medicinal and edible plant in Asia, particularly in Northeast Korea, Japan, China, and Russia (Lee et al., 2002; Liu et al., 2021). A member of the Araliaceae family, it is a shrub that can grow 2–7 m tall. *E. senticosus* leaves contain over three hundred compounds, mainly phenols, flavonoids, triterpenoid saponins, and polysaccharides, which contribute to their antioxidant activity and potential health benefits, such as anti-Alzheimer’s, anti-diabetic, and anti-obesity effects (Kwon et al., 2024). While dried roots, stem bark, and branch bark of *E. senticosus* are recognized medicinal parts traditionally used as tonics and sedatives for rheumatism, fatigue, and metabolic issues, the leaves have historically received less focus despite their rich phytochemical content (Shi et al., 2024). In Western pharmacopeias, medicinal applications mainly involve the roots, whereas East Asian traditions also include the leaves as vegetables, teas, and remedies for neurasthenia and overall wellness (Shi et al., 2024). Importantly, *E. senticosus* leaves are a plentiful but underused biomass resource. Unlike roots, which often damage or destroy the plant during harvest, leaves can be collected annually without harming the tree, providing a renewable, sustainable source of bioactive compounds, reducing cultivation costs, and conserving plant populations. Consequently, using *E. senticosus* leaves offers an environmentally friendly and economically viable alternative to root-based materials for the development of functional foods and nutraceuticals. Solid-state fermentation (SSF) is a process in which microorganisms, primarily fungi, are grown on solid substrates with low moisture levels. This method facilitates efficient interactions between the substrate and microbes while consuming less energy than submerged fermentation (Krishna, 2005; Pandey, 2003). In addition to its efficiency, SSF facilitates biochemical changes in plant matrices that effectively modify, liberate, or generate various bioactive compounds (Chilakamarry et al., 2022; Pandey et al., 2000). *Rhizopus oligosporus*, a fungus widely used in SSF and the predominant starter culture for fermented soybeans, was chosen in this study for its potent enzymatic profile, which facilitates the breakdown of complex plant materials. During fermentation, various hydrolytic enzymes produced by the fungus can transform bound compounds into more accessible forms (Zhang et al., 2022). Recent studies have shown that SSF with *R. oligosporus* can increase the production or accumulation of specific metabolites, including γ-aminobutyric acid, L-carnitine, and polyphenols (Lee et al., 2020; Lim et al., 2023). Lim et al. (2023) fermented wild-simulated ginseng leaves with *R. oligosporus* and observed the synthesis of new L-carnitine, along with increased levels of bioactive compounds, such as squalene, phenols, and saponins. Additionally, minor ginsenosides rose by 32.0% to 343.0% after fermentation (Lim et al., 2023). Although *E. senticosus* and *Panax ginseng* both belong to *the Araliaceae* family, they have distinct phytochemical profiles. *P. ginseng* is characterized by a high content of dammarane-type triterpenoid saponins (ginsenosides), which are considered its principal bioactive constituents (Lim et al., 2023). In contrast, *E. senticosus* leaves are particularly rich in phenylpropanoid glycosides (eleutherosides), chlorogenic acids, other caffeoylquinic acid derivatives, lignans, coumarins, flavonoids, and phytosterols (Lee et al., 2002; Liu et al., 2021; Shi et al., 2024). These marked differences in chemical composition provide distinct metabolic substrates for fermenting microorganisms and thereby influence microbial growth, enzyme production, and biotransformation pathways. Because *E. senticosus* leaves contain abundant eleutherosides, chlorogenic acids, and other caffeoylquinic acid derivatives that are largely absent from *P. ginseng*, fermentation by *R. oligosporus* is expected to promote substrate-specific biotransformation reactions, including glycoside hydrolysis, phenolic acid conversion, and the release of bound phenolic compounds, resulting in a metabolite profile distinct from that reported for fermented ginseng leaves. As a result, the composition of fermentation-derived metabolites and the functional properties of the final products are strongly dependent on both the phytochemical characteristics of the substrate and the fermentation conditions, including the microbial strain, fermentation time, temperature, moisture, and oxygen availability (Kim et al., 2025a, 2025b; Lee et al., 2025). Therefore, the present study investigates substrate-specific metabolic responses and fermentation outcomes, rather than merely applying an established fermentation process to a different plant material. Most current research has concentrated on identifying the bioactive compounds of *E. senticosus* and their biological effects, leaving a gap in studies that concurrently analyze microbial growth, enzymatic activity, and bioactive compounds in *E. senticosus* leaves. This study investigates how SSF using *R. oligosporus* modulates the functional and metabolic profile of *E. senticosus* leaves. Investigations focus on physical functionalities (water-holding, water-swelling, oil absorption, and cholesterol-binding capacities), bioactive constituents (total phenolic and total flavonoid contents, chlorogenic acid, caffeic acid, quinic acid, β-sitosterol, and L-carnitine), antioxidant efficacy determined through multiple radical scavenging and reducing power assays (ABTS, ORAC, FRAP, and TAC), and anti-inflammatory potential, assessed by quantifying the inhibition of nitric oxide (NO) production in lipopolysaccharide (LPS)-stimulated RAW 264.7 macrophages.

## Materials and methods

### Materials

*E. senticosus* leaves were gifted by a local farm in Pyeongchang (Gangwon-do, Republic of Korea). L-Carnitine ((3-hydroxy-4-*N*-trimethylaminobutyrate), ergosterol, and β-sitosterol were purchased from Tokyo Chemical Industry Co., Ltd. (Tokyo, Japan). Quercetin, gallic acid, ammonium formate, Trolox (6-hydroxy-2,5,7,8-tetramethylchroman-2-carboxylic acid), ABTS, *o*-phthaldehyde, 4-methylumbelliferyl β-d-glucopyranoside, chlorogenic acid, and quinic acid were obtained from Sigma-Aldrich (St. Louis, MO, USA). Dimethyl sulfoxide (DMSO), methanol, ethanol, acetonitrile, and ethyl acetate were purchased from Duksan Reagents Co., Ltd. (Ansan-si, Gyeonggi-do, Korea).

### Solid-state fermentation of *Eleutherococcus senticosus*

For spore production, *R. oligosporus* KCCM 11948P was grown on potato dextrose agar (PDA; Difco, Detroit, MI, USA). The plates were maintained at 30 °C for 5 days until sufficient sporulation was achieved. *E. senticosus* leaves were ground prior to fermentation. For each batch, 5 g of ground leaf sample was placed in a sterile Petri dish, and 10 mL of distilled water was added to adjust the moisture content. The samples were allowed to hydrate at room temperature for 12 h. After hydration, the samples were autoclaved at 121 °C for 15 min and then cooled to room temperature under sterile conditions. The sterilized samples were inoculated with *R. oligosporus* spores at a concentration of 5 × 10^5^ spores/g sample. The inoculated samples were incubated at 30 °C and 60% relative humidity for up to 120 h. Fermentation was carried out in independent batches, and samples were collected at 24 h intervals. At each time point, triplicate samples were harvested by removing individual Petri dishes and immediately stored at − 20 °C. After fermentation, both non-fermented and fermented samples were freeze-dried until a constant weight was achieved to remove residual moisture.

### Extraction of the sample

Aqueous extraction of either fermented or non-fermented *E. senticosus* leaves was performed by mixing 1 g of sample with 30 mL of distilled water. Ultrasonic-assisted extraction was carried out at 40 W for 30 min (Korea BioTech, Seongnam, Republic of Korea), followed by a thermal treatment (80 °C, 5 min). After centrifugation (9000×*g*, 20 min, 4 °C), the collected supernatant was passed through Whatman No. 1 filter paper. The filtrate was then frozen and lyophilized under vacuum conditions (− 45 °C, 10 Pa) to obtain the final powdered extract.

### Analysis of physical properties

#### Water holding capacity (WHC)

The sample (50 mg) was dispersed in 1 mL of deionized water and hydrated for 24 h at 22 °C (Ryu et al., 2024). Subsequently, the suspension underwent centrifugation at 10,000×*g* for 15 min. After the supernatant was discarded, the weight of the water retained by the pellet was measured. The WHC was then determined using the following formula:

$$WHC (g/g)=\frac{{W}_{1}-{W}_{2}}{{W}_{1}}$$

where W₁ is the initial dry sample weight (g), and W₂ is the weight of the hydrated residue (g).

#### Water swelling capacity (WSC)

WSC was analyzed by adding 50 mg of the sample (W₁) with 1 mL of deionized water, mixing well, and allowing hydration at 4 °C for 24 h (Ryu et al., 2024). The initial volume (*V₁*) and final swollen volume (*V₂*) were recorded and WSC was calculated as:

$$WSC (mL/g)=\frac{{V}_{1}-{V}_{2}}{{W}_{1}}$$

#### Oil adsorption capacity (OAC)

OAC was measured by mixing 50 mg of the sample with 1 mL of olive oil and incubating at room temperature for 24 h. The mixture was centrifuged at 10,000×*g* for 15 min, and the supernatant was removed. The residue was weighed. OAC was calculated as:

$$OAC (g/g)=\frac{{W}_{1}-{W}_{2}}{{W}_{1}}$$

here W₁ is the dry sample weight (g), and W₂ is the weight of the oil-adsorbed residue (g).

#### Cholesterol binding capacity (CBC)

For CBC analysis, sample (200 mg) was added to 10% (v/v) egg yolk solution (5 mL) adjusted to pH 2.0 or 7.0 and incubated at 37 °C with gentle agitation (120 rpm) for 2 h. The reaction mixture was mixed with four volumes of 95% ethanol and centrifuged at 3800×*g* for 20 min. The supernatant was collected, and ethanol was removed by evaporation. Cholesterol content was determined by reacting the extract with glacial acetic acid, 0.05% (w/v) *o*-phthalaldehyde, and concentrated sulfuric acid (1:1:1:5, v/v/v/v), followed by incubation at 25 °C for 20 min. Absorbance was measured at 550 nm using a microplate reader (SpectraMax M3, Molecular Devices, USA). CBC was expressed as mg of cholesterol bound per g of sample (mg/g).

### Phytochemical analysis

#### Ergosterol analysis

Ergosterol was extracted from both fermented and non-fermented *E. senticosus* leaves by alkaline saponification. Briefly, 300 mg of each sample was mixed with 10 mL of 10% (w/v) KOH in methanol and heated at 80 °C for 60 min with occasional stirring to ensure complete saponification. After cooling to room temperature, 3 mL of distilled water and 4 mL of hexane were added to the mixture. The solution was vortexed and allowed to stand until phase separation occurred. The upper hexane phase, containing ergosterol, was recovered. This extraction process was repeated twice using fresh 4 mL portions of hexane. The pooled hexane extracts were then evaporated using nitrogen gas, redissolved in methanol, and filtered through a 0.2 µm syringe filter. Ergosterol analysis was performed using a UPLC system (Waters, Milford, MA, USA) equipped with a BEH C_18_ column (2.1 × 100 mm, 1.7 µm) and a photodiode array detector. The system used isocratic elution with acetonitrile as the mobile phase at a flow rate of 0.1 mL/min for 11 min. Ergosterol (0.1–60 µg/mL in methanol) was used as the standard.

#### L-carnitine analysis

To analyze bioactive compounds in both non-fermented and fermented *E. senticosus* leaves, including L-carnitine, caffeic acid, quinic acid, chlorogenic acid, and total phenolic and flavonoid content, the stock solution was prepared by adding 10 mg of extracted powders of *E. senticosus* leaves to 1 mL of DMSO and dissolved well. For L-carnitine measurement, the stock was diluted with methanol to 1 mg/mL and filtered through a 0.2 µm syringe filter. The L-carnitine content was measured using a BEH HILIC column (2.1 × 100 mm, 1.7 µm; Waters, Milford, MA, USA) coupled to a mass spectrometer, according to our previous methodology (Lim et al., 2023). Standards for L-carnitine ranged from 0.002 to 2.0 µg/mL. To determine the limit of detection (LOD) and limit of quantification (LOQ) of the UPLC-MS method, ten L-carnitine standard solutions ranging from 0.002 to 2000 ng/mL were prepared. Three independent calibration curves were generated by analyzing each standard solution in triplicate. The standard deviation of the y-intercept (σ) and the mean slope (S) of the calibration curves were calculated and used to determine the LOD and LOQ according to the following equations. The LOD was calculated as LOD = 3.3σ/S, and the LOQ was calculated as LOQ = 10σ/S. The three independent calibration curves showed good linearity (R^2^ = 0.99), and the LOD and LOQ were determined to be 4.3 ng/mL and 13.0 ng/mL, respectively (Table S1).

#### Analysis of chlorogenic acid, caffeic acid, and quinic acid

The levels of caffeic acid and chlorogenic acid in both non-fermented and fermented *E. senticosus* leaves were measured using UPLC–MS (Waters UPLC system, Milford, MA, USA) coupled with a quadrupole mass spectrometer) in negative electrospray ionization mode (ESI⁻), targeting m/z 355.00 (chlorogenic acid), m/z 181.10 (caffeic acid), and m/z 191.00 (quinic acid). The BEH C_18_ column (2.1 × 100 mm, 1.7 µm) was used for analysis at a flow rate of 0.3 mL/min. The mobile phase included acetonitrile with 0.1% formic acid (solvent A) and water with 0.1% formic acid (solvent B). A gradient elution program was used: 2% A (0–3 min), 10% A (4 min), 15% A (5 min), increasing to 50% A (15 min), then 100% A (16–18 min), and finally returning to 2% A (18.1–20 min) for column re-equilibration. Chlorogenic acid and caffeic acid (0.05–100 µg/mL) were used as standards.

#### Analysis of β-sitosterol

For β-sitosterol analysis, 50 mg of freeze-dried extract of both non-fermented and fermented *E. senticosus* leaves was dissolved in ethyl acetate and vortexed. Then, 1 µL of the sample was spotted onto silica gel 60 F₂₅₄ plates (Supelco, Bellefonte, PA, USA), and the plate was developed by a single ascent in toluene/ethyl acetate/acetic acid (8:2:0.2, v/v/v). β-sitosterol was visualized by dipping the TLC plate into p-anisaldehyde–sulfuric acid reagent, followed by heating at 100 °C for 5 min. The amount of β-sitosterol was quantified by converting the integrated density into a standard curve using the AlphaEaseFC 4.0 Image Program (Alpha Inotech, San Leandro, CA, USA).

#### Total phenolic (TPC) and total flavonoid (TFC) content analysis

TPC of both non-fermented and fermented *E. senticosus* leaves was measured using the Folin–Ciocalteu method, as in our previous study (Kim et al., 2023), with gallic acid as the standard (1–100 µg/mL). Total flavonoid content (TFC) was measured using the aluminum chloride colorimetric method, as previously described (Chang et al., 2002), with quercetin (1–100 µg/mL) as the standard.

### Analyses of antioxidant properties

#### ABTS assay

The ABTS radical-scavenging activity of non-fermented and fermented *E. senticosus* leaves was assessed according to (Re et al., 1999). The ABTS•⁺ reagent was prepared by mixing 7 mM ABTS with 2.45 mM potassium persulfate and allowing the mixture to react in the dark at room temperature. After 14 h, the resulting ABTS•⁺ solution was diluted with 75 mM sodium phosphate buffer (pH 7.4) to reach an absorbance of 0.70 ± 0.03 at 734 nm, using a SpectraMax M3. The diluted reagent was then used for the assay. For the test, 20 µL of the sample was mixed with 180 µL of ABTS solution in a 96-well plate, then incubated at 22 °C for 30 min. The plate was read at 734 nm with a SpectraMax M3. A reaction containing only ABTS used as the control. The ABTS•⁺ radical-scavenging activity was calculated relative to the control.

#### Ferric reducing antioxidant power (FRAP) assay

The FRAP assay of non-fermented and fermented *E. senticosus* leaves was performed following Lim et al. (2022). The FRAP working solution was created by mixing 0.3 M acetate buffer (pH 3.6), 10 mM TPTZ in 40 mM HCl, and 20 mM FeCl₃·6H₂O in a 10:1:1 (v/v/v). The reaction mixture included 6.25 µL of sample or standard (FeSO₄·7H₂O, 10–2500 µM), 18.75 µL of water, and 187.5 µL of FRAP reagent in a 96-well plate. The plate was incubated at 22 °C in the dark for 30 min, then measured at 593 nm using SpectraMax M3. FRAP results were expressed as mM Fe2⁺ per gram of dry mass (mM Fe2⁺/g DM).

#### Total antioxidant assay

Total antioxidant activity of *E. senticosus* leaves was measured using the phosphomolybdenum method with minor modifications (Saeed et al., 2012). The reaction solution composed of 28 mM sodium phosphate, 4 mM ammonium molybdate, and 0.6 M sulfuric acid. To perform the antioxidant assay, 100 µL of sample or Trolox standard (0–500 µM) was added to 1 mL of reaction solution, then incubated at 95 °C for 90 min. Following cooling to 22 °C, a 200 µL aliquot of the reaction mixture was transferred into a 96-well microplate. The plate was then recorded at 695 nm employing a SpectraMax M3 reader. Total antioxidant capacity was quantified as mM Trolox equivalents (TE) per gram of dry matter (mM TE/g DM).

#### Oxygen radical absorbance capacity (ORAC) assay

The ORAC assay of both non-fermented and fermented *E. senticosus* leaves was conducted following (Ou et al., 2001). Fluorescein (25 nM) and AAPH (25 mM) were prepared in 75 mM sodium phosphate buffer at pH 7.4. For the procedure, 10 µL of the sample or Trolox standard (ranging from 0 to 300 µM), 90 µL of fluorescein solution, and 100 µL of AAPH solution were sequentially added to a 96-well plate. The plate was then incubated at 37 °C for 120 min, with excitation and emission wavelengths of 485 and 538 nm, respectively, and measurements were taken every 3 min. The results were calculated based on the area under the fluorescence decay curve and expressed as Trolox equivalents per gram of dry mass (mM TE/g DM).

### Cell viability test

Cell viability of non-fermented and fermented *E. senticosus* leaves in RAW264.7 macrophages was assessed using our previously described methods (Kim et al., 2024). In brief, cells were inoculated into 96-well plates at a density of 2 × 10^4^ cells/well and maintained at 37 °C in a 5% CO₂ atmosphere. Following a 24 h incubation, the cells were exposed to various sample concentrations (200–800 µg/mL) for an additional 24 h. Untreated cells served as the control group. Then, the culture medium was replaced with a mixture of 90 µL DMEM and 10 µL Ez-CyTox reagent, followed by incubation for 1 h at 37 °C. The plate was read at 450 nm using a SpectraMax M3 reader.

### Inhibition of nitric oxide production

RAW 264.7 macrophages were plated in 96-well microplates at a density of 2 × 10^4^ cells/well and maintained in a humidified incubator (37 °C, 5% CO_2_). Following a 24-h adherence period, the culture media were replaced with fresh media containing various sample concentrations (200–800 µg/mL) and lipopolysaccharide (LPS, 1 µg/mL). Cells stimulated with LPS in the absence of samples served as the control. After a subsequent 24 h incubation, nitric oxide (NO) production was quantified by reacting the culture supernatants with Griess reagent in a 1:1 (v/v) ratio for 20 min at 22 °C. The plate was then recorded at 540 nm using a SpectraMax M3 reader. The suppressive effects of fermented and non-fermented *E. senticosus* extracts on NO release were determined relative to the control.

### Statistical analysis

Data are expressed as mean ± standard deviation (SD) (n = 3). Statistical analyses were analyzed by using SPSS software (version 29.0; IBM Corp., Armonk, NY, USA). Differences among groups were evaluated by one-way analysis of variance (ANOVA) followed by Tukey’s multiple comparison test. A value of p < 0.05 was considered statistically significant. The multivariate statistical analysis, including principal component analysis (PCA), Pearson correlation analysis, and heatmaps, was analyzed using OriginPro 2022b (OriginLab, Northampton, MA, USA).

## Results and discussion

### The effect of solid-state fermentation on the physical properties of fermented *E. senticosus* leaves

#### Water holding (WHC), water-swelling (WSC), and oil absorption (OAC) capacity

**Fig. 1:**
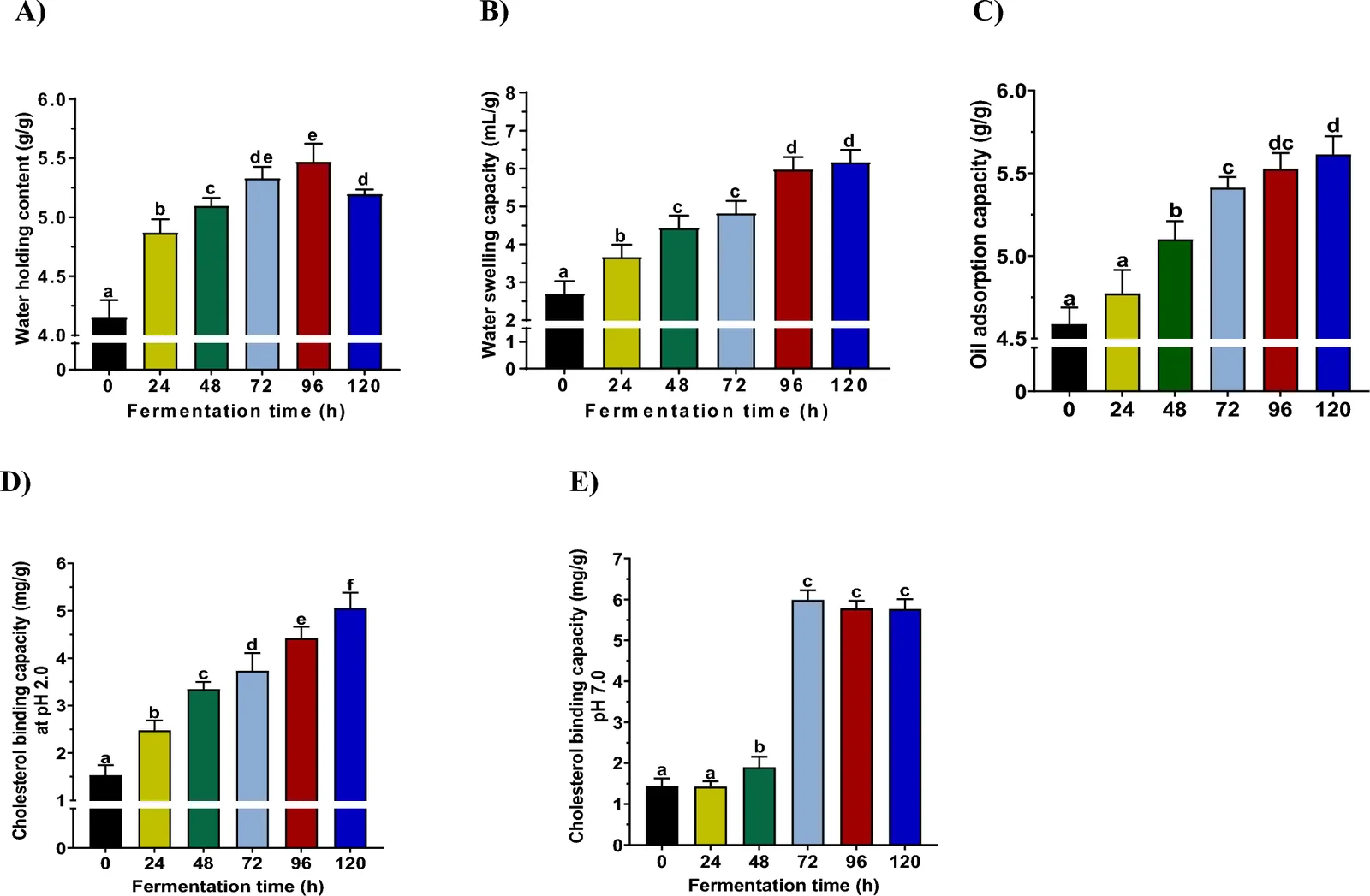
Physical characterization, including water holding capacity (**A**), water swelling capacity (**B**), oil absorption capacity (**C**), and cholesterol binding capacity at pH 2.0 (**D**) and pH 7.0 (**E**) of fermented *E. senticosus* leaves using *R. oligosporus* KCCM 11948P. Data are expressed as mean ± standard deviation (n = 3). Different letters denote significant differences identified through Tukey’s multiple-comparison test (p < 0.05)

WHC, WSC, and OAC are key parameters of dietary fiber that indicate its water-retention, swelling, and oil-absorption abilities during hydration. For example, dietary fiber's ability to retain oil can help prevent fat loss during cooking, while its capacity to adsorb or bind bile acids and promote their excretion is associated with lowering plasma cholesterol (Tosh and Yada, 2010). These are important for both physiological roles and food system applications. The values of WHC, WSC, and OAC for non-fermented and fermented *E. senticosus* leaves are shown in Fig. 1(A)–(C). The WHC of *E. senticosus* leaves rose from 4.15 ± 0.15 g/g to 5.47 ± 0.16 g/g, representing an increase of 31.8%, as fermentation time increased from 0 to 96 h. It then slightly declined to 5.19 ± 0.04 g/g [Fig. 1(A)]. The WSC increased from 2.70 ± 0.33 mL/g to 6.16 ± 0.33 mL/g over fermentation from 0 to 120 h, representing an increase of 128.1%. No significant differences in WSC were observed between 48- and 72 h, or between 96- and 120 h, of fermentation [Fig. 1(B)]. The OAC increased from 4.59 ± 0.10 g/g in the non-fermented leaves to 5.61 ± 0.11 g/g after 120 h of fermentation [Fig. 1(C)], showing an increase of 22.2%. Using the same *R. oligosporus* KCCM11948P for Maca fermentation, Ruy et al. (2024) reported similar WHC, WSC, and OAC results. The increase in WHC and WSC of *E. senticosus* leaves after fermentation with *R. oligosporus* resulted from microstructural changes in the leaves, which loosened their structure and degraded insoluble fibers and polysaccharides. This process created space within the molecular structure and exposed hydrophilic groups, leading to higher WHC and WSC (Ma et al., 2023; Sun et al., 2020). The exposed functional groups also helped physically trap oil within the matrix and improved OAC (Jia et al., 2019; Lin et al., 2022).

#### Cholesterol binding capacity (CBC)

CBC is recognized for its ability to reduce serum cholesterol and lower cardiovascular disease risk. The CBC for non-fermented and fermented *E. senticosus* leaves at pH 2.0 (representing an in vitro gastric environment) and pH 7.0 (representing an in vitro small-intestinal environment) are shown in Fig. 1(D) and (E), respectively. At pH 2.0, the CBC increased steadily during fermentation, showing an increase of 230.7% after 120 h [Fig. 1(D)]. In contrast, at pH 7.0, the CBC remained stable for the first 24 h before rising by 318.2% at 72 h [Fig. 1(E)]. There were no significant differences in CBC between 0 and 24 h of fermentation, nor among 72, 96, and 120 h. Similar trends were observed in fermented maca using *R. oligosporus*, where CBC was higher at pH 7.00 than at pH 2.0. CBC in maca increased continuously at pH 2.0, whereas at pH 7.0 it rose until 72 h and then stabilized through 120 h of fermentation (Ryu et al., 2024). Unlike the root and stem, *E. senticosus* leaves contain triterpenoid saponins (Adhimoolam et al., 2024) that can lower serum cholesterol levels by forming large, insoluble complexes with cholesterol (Sharmin et al., 2021). Therefore, the increase in CBC of fermented *E. senticosus* leaves may be associated with the saponin levels, but further research is required to clarify changes in saponin content during solid-state fermentation.

### Effects of SSF on biochemical properties of *E. senticosus* leaves

#### Ergosterol content

**Fig. 2:**
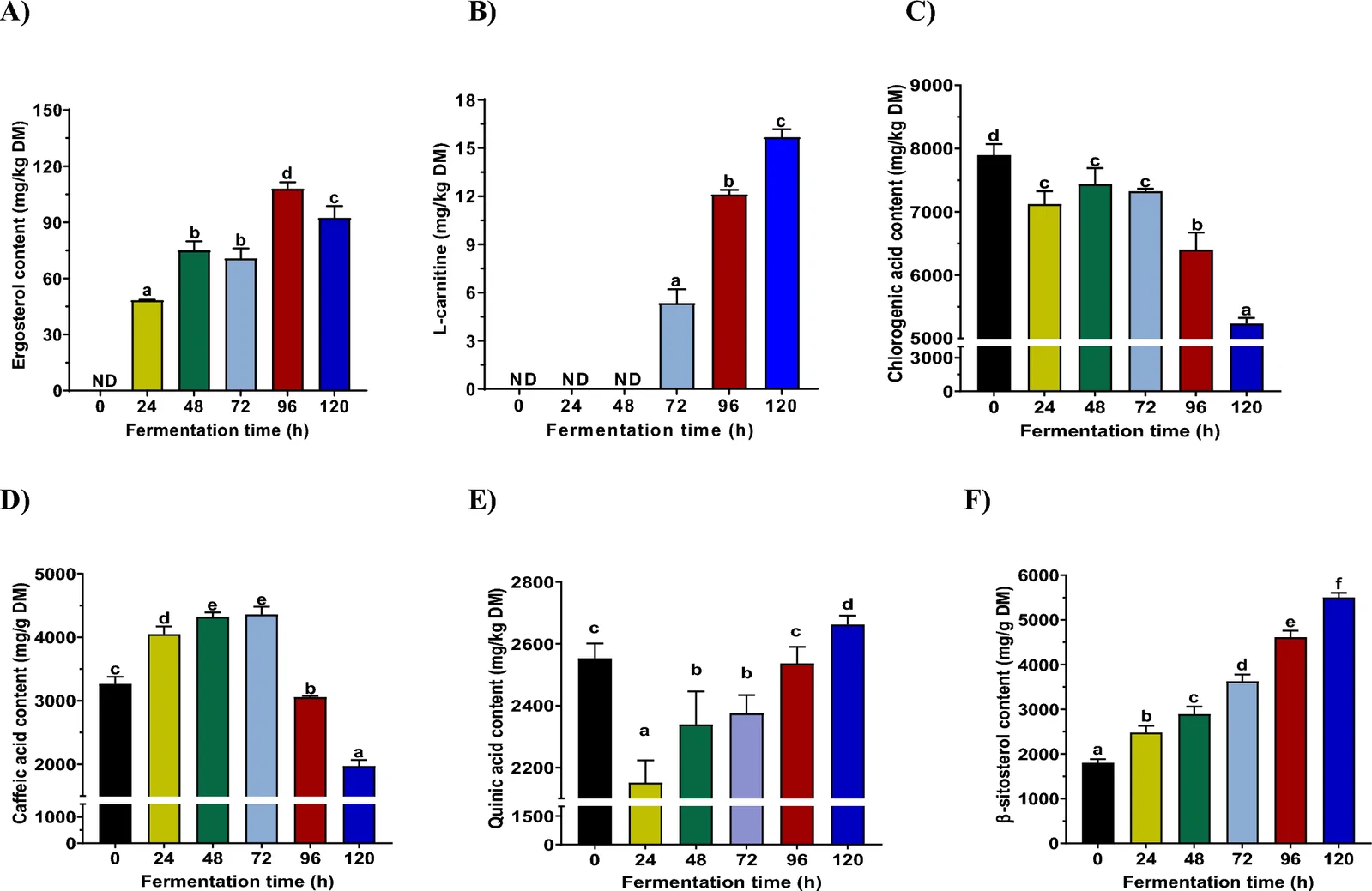
Effects of solid-state fermentation on the contents of ergosterol (**A**), L-carnitine (**B**), chlorogenic acid (**C**), caffeic acid (**D**), quinic acid (**E**), and β-sitosterol (**F**) in *E. senticosus* leaves. Data are presented as the mean ± standard deviation (n = 3). Different letters indicate significant differences according to Tukey’s multiple-comparison test (p < 0.05)

Ergosterol (5,7-diene cholesterol) is an important biomarker present in the cell membranes of most fungi and is used to assess fungal growth during SSF (Liu et al., 2024; Steudler et al., 2015). Thus, the growth of *R. oligosporus* on *E. senticosus* leaves during solid-state fermentation, as indicated by ergosterol levels, is shown in Fig. 2(A). Ergosterol was detected in the leaves after 24 h of fermentation, increased up to 48 h, remained steady through 72 h, rose again at 96 h, and then declined after 120 h. The highest ergosterol content in *E. senticosus* leaves (107.92 ± 3.52 mg/kg DM) was observed after 96 h of fermentation [Fig. 2(A)].

#### L-carnitine content

Synthesized from methionine and lysine, L-carnitine plays a pivotal role in energy metabolism. It primarily shuttles long-chain fatty acids into the mitochondria for β-oxidation. Additionally, this compound regulates the mitochondrial acyl-CoA/CoA ratio, enhances the oxidation of branched-chain amino acids, and serves as a vital component of the body’s internal antioxidant system (Elantary and Othman, 2024; Virmani and Cirulli, 2022). While approximately 25% of L-carnitine is produced endogenously in the liver, kidneys, and brain, the majority (75%) is derived from the diet (Reuter and Evans, 2012). Red meat is a rich source of L-carnitine; however, plant-derived materials generally contain low concentrations, highlighting the (Zhang et al., 2022) need for alternative enrichment strategies (Pekala et al., 2011). L-carnitine in *E. senticosus* leaves remained undetectable during the initial phases of fermentation (0–48 h). It was first identified at 72 h, reaching a concentration of 5.34 ± 0.87 mg/kg DM. Subsequently, the levels rose progressively, peaking at 15.67 ± 0.50 mg/kg DM by the end of the 120-h fermentation [Fig. 2(B)]. In agreement with previous studies, *R. oligosporus* facilitated L-carnitine synthesis during the SSF process (Hur et al., 2018; Lim et al., 2023; Ryu et al., 2024). Compared with other substrates after 5 days of fermentation, the L-carnitine content in fermented *E. senticosus* leaves was 917.5% higher than that in fermented quinoa but only 9.5% of that in fermented wild-simulated ginseng leaves. The detection of L-carnitine in *E. senticosus* leaves after fermentation with *R. oligosporus* indicates activation of a conserved fungal biosynthetic pathway. During fermentation, *R. oligosporus* secretes extracellular enzymes, including proteases, endoglucanases, exoglucanases, β-glucosidases, and esterases, which degrade plant macromolecules and enhance the availability of nutrients. In particular, protease activity releases amino acids, including lysine and methionine, which serve as essential precursors for L-carnitine biosynthesis (Ryu et al., 2024; Zhang et al., 2022). Zhang et al. (2022) reported that solid-state fermentation (SSF) of soybeans for over 60 h resulted in protease levels exceeding 40 U/g, thereby increasing the concentrations of soluble proteins and low-molecular-weight peptides. Similarly, *R. oligosporus* cultivated on unpeeled cassava tubers exhibited a robust protease activity of 1972.6 U/mg of protein (Egbune et al., 2023). Ryu et al. (2024) found that fermenting maca with *R. oligosporus* increased methionine levels from 0.48 to 1.32 mg/g and leucine from 3.33 to 5.78 mg/g. Fungi synthesize L-carnitine from L-lysine and L-methionine via a conserved four-step pathway involving ε-N-trimethyllysine (TML). In *Neurospora crassa*, free lysine is methylated three times by S-adenosyl-L-methionine to produce TML, with methionine providing the methyl groups. TML is then converted into β-hydroxy-TML by TML hydroxylase, further broken down into γ-trimethylaminobutyraldehyde, oxidized to γ-butyrobetaine, and finally hydroxylated into L-carnitine by γ-butyrobetaine hydroxylase (Swiegers et al., 2002). Initially identified in *N. crassa*, this pathway now serves as a key model for studying L-carnitine biosynthesis in eukaryotes and likely contributes to the elevated L-carnitine levels observed during the solid-state fermentation of *E. senticosus* leaves.

#### Chlorogenic, caffeic, and quinic acid content

The levels of chlorogenic, caffeic, and quinic acids in *E. senticosus* leaves are illustrated in Fig. 2(C)–(E). Chlorogenic acid dropped from 7894.08 ± 175.74 mg/kg DM at 0 h to 7120.43 ± 205.12 mg/kg DM at 24 h, rose to 7437.46 ± 252.93 mg/kg DM at 48 h, and continuously declined to 5233.53 ± 90.77 mg/kg DM at 120 h [Fig. 2(C)]. Caffeic acid peaked at 72 h of fermentation, increasing by 33.5% over non-fermented leaves. There was no significant difference in caffeic acid content between 48 and 72 h. After 72 h, it decreased to 1969.32 ± 98.35 mg/kg DM, which is 39.7% lower than that of non-fermented *E. senticosus* leaves [Fig. 2(D)]. Quinic acid sharply declined from 2552.75 ± 48.66 mg/kg DM at 0 h to 2150.75 ± 72.78 mg/kg DM after 24 h, then increased again to 2661.95 ± 29.3 mg/kg DM after 120 h, representing an increase of 4.3% compared to non-fermented *E. senticosus* leaves [Fig. 2(E)]. Fungal fermentation reliably breaks down chlorogenic acids in coffee. For example, Lee et al. (2016) found an 18.96% decrease in chlorogenic acid in green coffee beans after fermentation with *Rhizopus oligosporus* (Lee et al., 2016). Similarly, Arslan-Tontul et al. (2025) observed comparable degradation in coffee grounds undergoing SSF with *Aspergillus awamori*. Conversely, the metabolite caffeic acid shows more complex behavior; Kwak et al. (2023) reported that caffeic acid levels varied over a 10-day period, increasing until day 5, then decreasing by day 7, and rising again on day 10 (Kwak et al., 2023). Chlorogenic acid is an ester formed between caffeic acid and quinic acid. During fermentation, *R. oligosporus* secretes extracellular esterases that hydrolyze chlorogenic acid into caffeic acid and quinic acid, thereby increasing the caffeic acid content in *E. senticosus* leaves during the early stages of fermentation (up to 72 h) [Fig. 2(D)] (Zhang et al., 2022). As fermentation progressed, the declining caffeic acid levels indicate that it served as a substrate for fungal metabolism. Lee et al. (2016) also reported substantial breakdown of caffeic acid during *R. oligosporus* fermentation of green coffee beans, which was associated with an approximately twofold increase in volatile phenolic derivatives. These findings suggest that caffeic acid functions as an intermediate metabolite, being converted into volatile phenolic compounds and other secondary metabolites during fungal fermentation rather than accumulating (Lee et al., 2016). Unlike caffeic acid, quinic acid first decreased and then increased during fermentation. The initial decline in quinic acid likely results from its rapid utilization by the fungus as a carbon source (Niu et al., 2017), whereas its subsequent increase during SSF mainly results from the continued esterase-mediated hydrolysis of chlorogenic acid. Similarly, Arancibia-Díaz et al. (2025) found that quinic acid levels continued to increase in the later stages of solid-state fermentation, peaking after prolonged fungal activity, while chlorogenic acid decreased due to enzymatic hydrolysis (Arancibia-Diaz et al., 2025). These observations imply that the final quinic acid concentration depends on the balance between its release from chlorogenic acid and microbial consumption. The different timing patterns of these compounds reveal a dynamic reorganization of the phenolic metabolic pathway during SSF with *R. oligosporus*. These results indicate that the observed phenolic changes are governed by the coordinated activities of fungal esterases and downstream metabolic pathways, rather than by simple chemical degradation of chlorogenic acid.

#### β-sitosterol content

Β-sitosterol is the major phytosterol in most plants, with a structure similar to cholesterol but with an additional ethyl group. It supports human health by reducing the absorption of low-density lipoprotein, also known as "bad” cholesterol (Adhimoolam et al., 2024). Since humans cannot produce β-sitosterol themselves, it must be acquired through diet as a micronutrient. Therefore, β-sitosterol in fermented *E. senticosus* leaves was investigated. The results are shown in Fig. 2(F). β-sitosterol exhibited a consistent and significant rise throughout the fermentation process. In fermented *E. senticosus* leaves with *R. oligosporus*, its content increased by 205.2% after 120 h. In this study, β-sitosterol was identified in the water-soluble extract of *E. senticosus* leaves. The stems and roots of *E. senticosus* mainly contain phenylpropanoids, lignans, and coumarins, while the fruits are high in polyphenols, flavonoids, and essential oils. In contrast, the leaves are rich in triterpenoid saponins, flavonoids, caffeoylquinic acids, and polysaccharides (Adhimoolam et al., 2024). The presence of β-sitosterol in the water extract of *E. senticosus* leaves may be attributed to saponins, which can increase the water solubility of hydrophobic compounds by forming micelles (Kim et al., 2024; Nicol et al., 2017). *R. oligosporus* also produces a range of enzymes, including cellulase, glucanase, xylanase, amylase, protease, esterase, lipases, and β-glucosidase (Lim et al., 2023; Sivaramakrishnan et al., 2021; Zhang et al., 2022). These enzymes break down the oilseed cell wall, releasing oil bound to proteins and polysaccharides, forming lipoproteins or lipopolysaccharides, or aiding in the formation of sterol esters from sterols and fatty acids in the fermentation medium (Dulf et al., 2015). Dulf et al. (2016) observed a 500% increase in sterol esters in plum kernels during solid-state fermentation with *R. oligosporus* (Dulf et al., 2016). However, further study is required on the water solubility of β-sitosterol, as well as its increase by enzymes during SSF.

### Total phenolic (TPC) and flavonoid (TFC) content of *E. senticosus* leaves

**Fig. 3:**
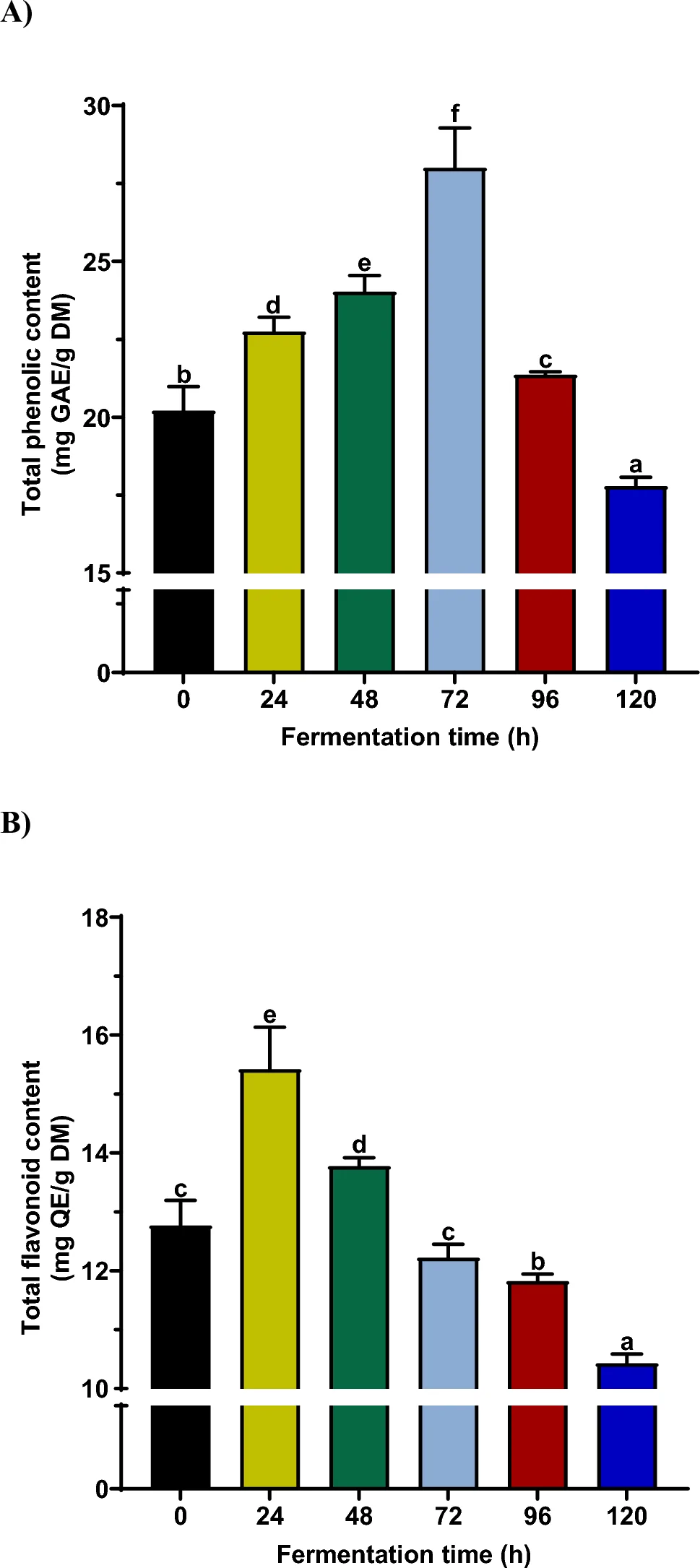
Total phenolic content (**A**) and total flavonoid content (**B**) of *E. senticosus* leaves fermented with *R. oligosporus* KCCM 11948P. Data are presented as the mean ± standard deviation (n = 3). Different letters indicate significant differences according to Tukey’s multiple-comparison test (p < 0.05)

The total phenolic content (TPC) in *E. senticosus* leaves rose from 20.21 ± 0.77 mg GAE/g to 28.00 ± 1.27 mg GAE/g DM, an increase of 38.6% as fermentation time extended from 0 to 72 h [Fig. 3(A)]. After 120 h, it decreased to 17.79 ± 0.29 mg GAE/g DM. Similar trends were observed in fermented wild-simulated ginseng leaves with *R. oligosporus*, where TPC peaked on day 3 of fermentation, increasing by 43% (Lim et al., 2023). Kwak et al. (2023) noted that the TPC of Yerba mate increased by 20% after 1 day of fermentation before gradually declining up to day 10 (Kwak et al., 2023). Ryu et al. (2024) observed a slight increase in TPC of maca, reaching its maximum on day 5 of fermentation, which was 18.0% higher than non-fermented maca using the same *R. oligosporus* KCCM 11948P. TFC of *E. senticosus* leaves peaked after 24 h of fermentation, showing an increase of 20.8% compared to that of non-fermented *E. senticosus* leaves. Subsequently, it decreased as fermentation time increased from 48 to 120 h [Fig. 3(B)]. Phenolic compounds are mainly stored in a bound form within cell membranes, with only small amounts freely soluble. *R. oligosporus* produces hydrolytic enzymes such as cellulase, xylanase, β-glucosidase, pectinase, tannase, protease, lipase, phytase, and esterase, which degrade the plant cell wall matrix and hydrolyze ester- or glycosidic-bound phenolic compounds during SSF (Lim et al., 2023; Varzakas, 1998; Zhang et al., 2022, 2026). Consequently, phenolic acids and flavonoid aglycones that were previously inaccessible become liberated, resulting in an apparent increase in TPC and TFC. The decline of TPC of *E. senticosus* leaves after 72 h fermentation and TFC after 24 h of fermentation was possible caused by the utilization of phenolic compounds as secondary carbon sources or metabolize them via oxidative and catabolic pathways (Leonard et al., 2021). Phenolic acids may undergo decarboxylation, dehydroxylation, methylation, reduction, or ring-cleavage reactions catalyzed by fungal enzymes such as phenol oxidases, laccases, peroxidases, and dioxygenases, thereby decreasing the concentration of extractable phenolics. Likewise, flavonoids can be oxidized, polymerized, or further transformed into low-molecular-weight metabolites that are not efficiently quantified by conventional TPC and TFC assays. Furthermore, oxidative polymerization of phenolic compounds into insoluble, high-molecular-weight complexes reduces their extractability, contributing to the observed decline during the late stage of fermentation (Lee et al., 2016; Leonard et al., 2021).

### Antioxidant properties of fermented *E. senticosus* leaves

**Fig. 4:**
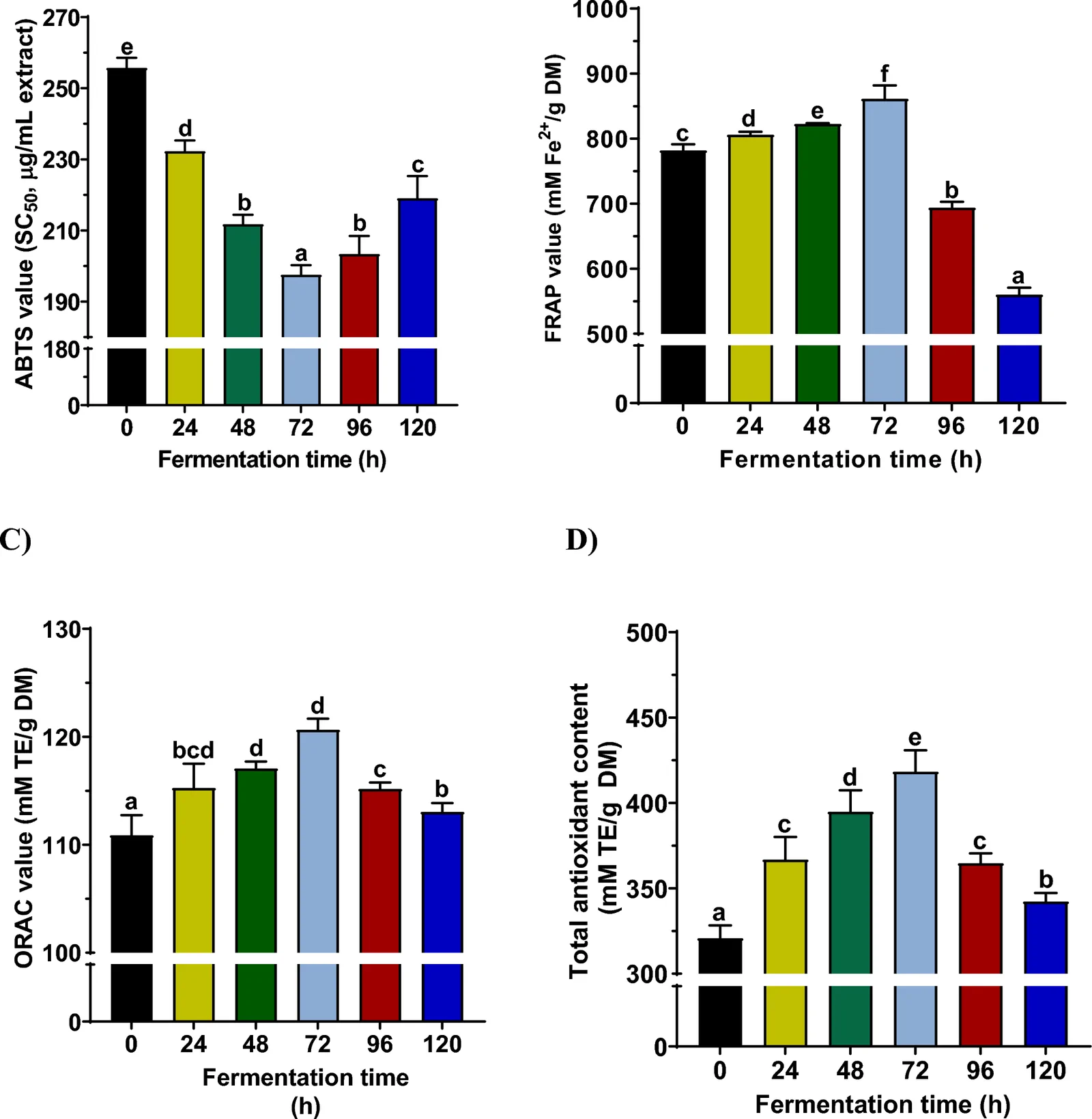
Changes in antioxidant capacity, including ABTS (**A**), FRAP (**B**), ORAC (**C**), and TAC (**D**) of *E. senticosus* leaves during solid-state fermentation using *R. oligosporus*. Data are expressed as mean ± standard deviation (n = 3). Different letters denote significant differences identified through Tukey’s multiple-comparison test (p < 0.05)

Antioxidants are bioactive compounds that suppress oxidative stress, thereby protecting cellular structures from free radical-induced damage. Consequently, increased intake of these compounds is associated with a reduced risk of chronic diseases, including cancer, Alzheimer's disease, and cardiovascular disorders (Chandimali et al., 2025). The changes in the antioxidant properties of *E. senticosus* leaves during 0 to 120 h of fermentation were assessed using two mechanisms: single-electron transfer (SET), including ABTS, FRAP, and TAA assays, and hydrogen-atom transfer (HAT), such as the ORAC assay. The results are presented in Fig. 4. The ABTS radical-scavenging activity was measured using the SC_50_ value, where a lower SC_50_ indicates stronger antioxidant activity. In contrast, FRAP, TAA, and ORAC results are expressed as Trolox equivalents (TE), with higher TE values indicating greater activity. The SC_50_ value of *E. senticosus* leaves decreased from 255.72 ± 2.85 µg/mL to 197.56 ± 2.70 µg/mL after 72 h of fermentation [Fig. 4(A)], showing a 22.7% reduction in SC_50_, indicating an improvement in antioxidant capacity. After 120 h, the SC_50_ value increased again to 219.04 ± 6.28 µg/mL [Fig. 4(A)]. The FRAP, TAA, and ORAC assays displayed similar patterns, with antioxidant levels peaking at 72 h and declining afterward toward 120 h [Fig. 4(B)–(D)]. Maximal antioxidant activities were observed after 72 h of fermentation, with an increase of 10.2%, 30.4%, and 8.8% in FRAP, TAA, and ORAC, respectively. In contrast, the antioxidant activities (ABTS, FRAP, ORAC, and TAA) of fermented *E. senticosus* leaves decreased after 72 h, aligning with reductions in several key antioxidant phytochemicals. Specifically, the total phenolic content (TPC) at 120 h dropped by 12.0% and 36.5% compared with the 0- and 72-h fermented *E. senticosus* leaves, respectively, while the total flavonoid content (TFC) decreased by 18.3% and 32.4% relative to the 0- and 24-h fermented *E. senticosus* leaves. Additionally, chlorogenic acid levels declined during fermentation, and caffeic acid also decreased after 72 h. These compositional changes are consistent with the observed reduction in antioxidant capacity, suggesting that prolonged fermentation may reduce the abundance of antioxidant phytochemicals. Although the underlying mechanism was not directly investigated in the present study, we hypothesize, based on previous reports, that prolonged fungal fermentation may promote microbial metabolism, oxidation, and biotransformation of phenolic compounds, thereby reducing their extractable levels and antioxidant activity (Correia et al., 2004; Lim et al., 2022; Sivaramakrishnan et al., 2021; Zhang et al., 2022). Future studies involving direct measurements of oxidative enzyme activities, such as polyphenol oxidase and laccase, are needed to verify this proposed mechanism. Our results align with the antioxidant effects observed in fermented wild-simulated ginseng leaves, Yerba mate, and maca with *R. oligosporus*. Lim et al. (2023) found that wild-simulated ginseng leaves reached peak antioxidant levels in ORAC and FRAP assays at 72 h of fermentation, then decreased by day 10. Yerba mate leaves exhibited the highest antioxidant activity after 1 day of fermentation, with ORAC and FRAP levels increasing by 92.0% and 9.0% greater than those of non-fermented samples, respectively. While ORAC values for Yerba mate gradually declined from days 3 to 10, its FRAP values remained high until day 7, then decreased on day 10 (Kwak et al., 2023). During fermentation, the contents of caffeic acid, β-sitosterol, total phenolic, and flavonoid contents increased by 33.5%, 205.2%, 38.6%, and 20.8%, respectively, due to hydrolytic enzymes produced during SSF. Additionally, L-carnitine, which is newly synthesized in *E. senticosus* leaves, not only facilitates the transport of long-chain free fatty acids into mitochondria but also exhibits strong antioxidant activity, as shown by in vitro tests such as FRAP, hydrogen peroxide scavenging, and DPPH (Gulcin, 2006). Therefore, the increase in phytochemicals probably accounts for the enhanced antioxidant activity observed in *E. senticosus* leaves during SSF.

### Cell viability and suppression of nitric oxide formation of fermented *E. senticosus* leaf

**Fig. 5:**
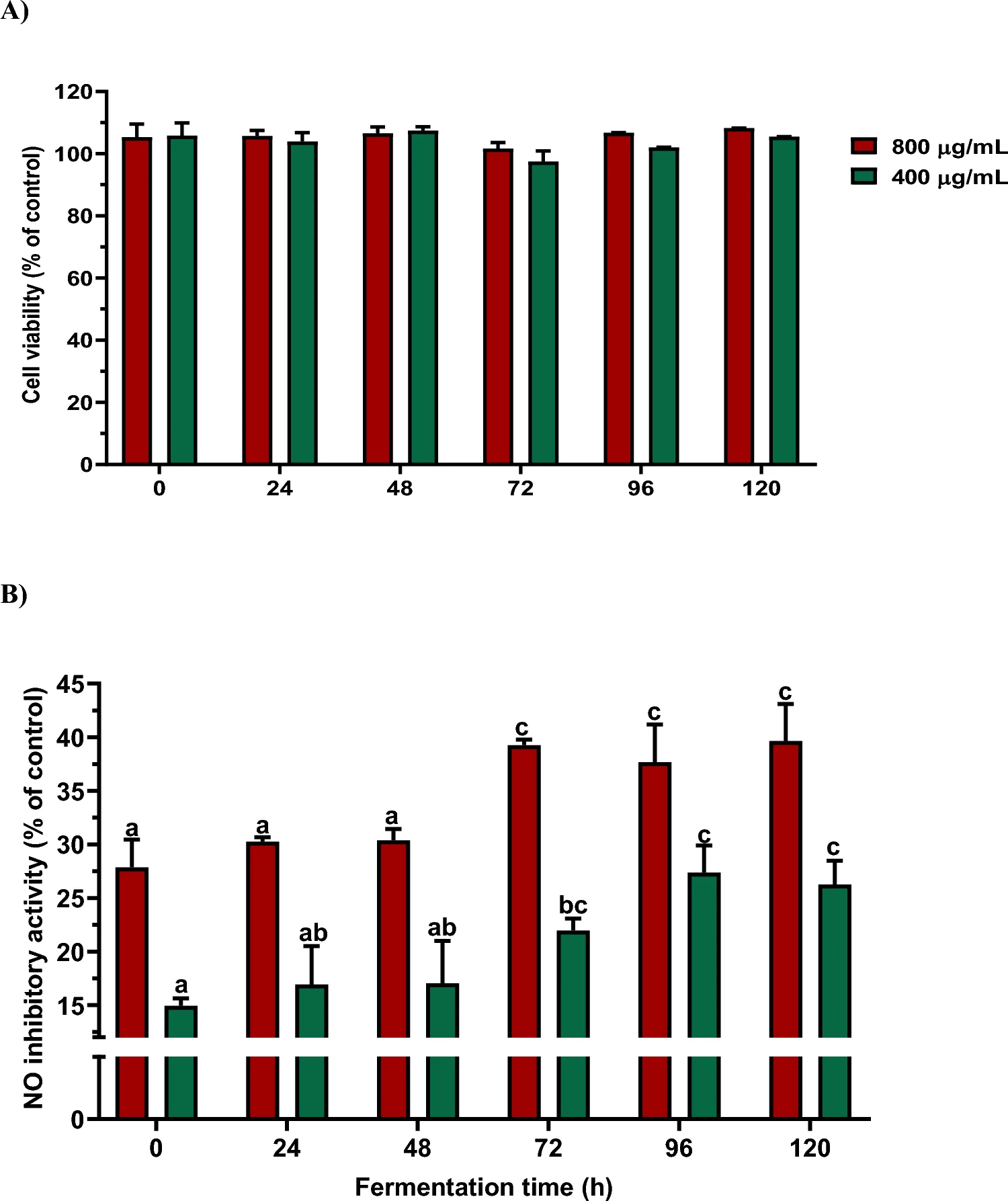
Cell viability (**A**) and suppression of nitric oxide production in LPS-simulated RAW264.7 cells (**B**) of *E. senticosus* leaves during solid-state fermentation using *R. oligosporus*. Data are expressed as mean ± standard deviation (n = 3). Different letters denote significant differences identified through Tukey’s multiple-comparison test (p < 0.05)

The cytotoxicity and NO-inhibitory activity of non-fermented and fermented *E. senticosus* leaves using *R. oligosporus* at concentrations of 400 µg/mL and 800 µg/mL on RAW264.7 macrophage cells were examined. The results are presented in Fig. 5. No cytotoxicity towards RAW264.7 macrophage cells was observed at the tested concentrations [Fig. 5(A)]. Consequently, these doses were used to evaluate their inhibitory effects on NO production in LPS-stimulated RAW264.7 macrophage cells. At 800 µg/mL, the NO-inhibitory activity of *E. senticosus* leaves showed no significant differences among fermentation times of 0, 24, and 48 h or among 72, 96, and 120 h. However, NO-inhibitory activity increased by 40.9% at 72 h of fermentation compared to non-fermented leaves [Fig. 5(B)]. At 400 µg/mL, the inhibitory activity followed similar patterns, with no significant differences among the same fermentation durations. The highest NO-inhibitory activity at 400 µg/mL was observed at 96 h, showing an increase of 83.2% over non-fermented leaves. These findings support previous studies indicating that *E. senticosus* leaves inhibit NO production in LPS-stimulated RAW264.7 macrophage cells (Shi et al., 2024). Additionally, Shi et al. (2024) reported that the anti-inflammatory effects of *E. senticosus* leaves involve reducing pro-inflammatory mRNA levels, including interleukin-1β, interleukin-6, nitric oxide synthase, and cyclooxygenase, in RAW264.7 cells.

### Correlation analysis

**Fig. 6:**
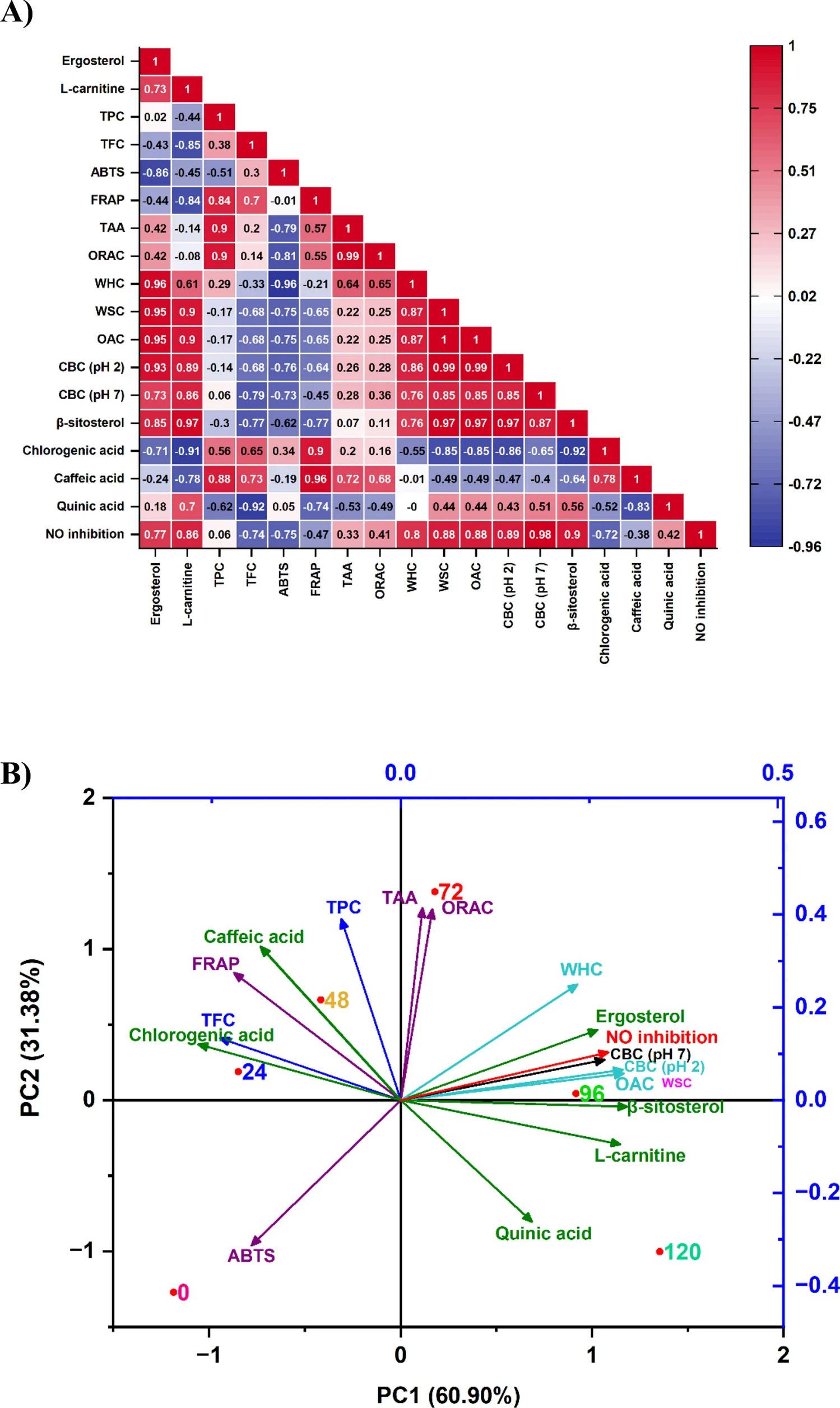
Pearson correlation heatmap showing enzymatic activity, phenolic content, antioxidant capacity, and metabolite profiles during solid-state fermentation of *A. senticosus* by *R. oligosporus*. The correlation coefficient (r) ranges from -1 (negative correlation) to + 1 (positive correlation), as indicated by the color scale

The dynamic relationships between the phytochemical compounds and functional properties of *E. senticosus* leaves over the 120 h of SSF using *R. oligosporus* were elucidated using Pearson correlation analysis [Fig. 6(A)]. Within the resulting heatmap matrix, the correlation coefficient (r) evaluates the linear interdependence of variables on a scale from − 1 to + 1, where positive values denote a synergistic trend and negative values indicate a reciprocal, inverse association. Antioxidant activity measured by ABTS correlated strongly negatively with ergosterol (r = − 0.86), while FRAP correlated negatively with L-carnitine (r = − 0.84) and positively with TPC (r = 0.84), chlorogenic acid (r = 0.9), and caffeic acid (r = 0.96) [Fig. 6(A)]. ORAC and TAA antioxidant measurements showed a positive correlation with TPC (r = 0.9). Physical properties such as WSC, OAC, and CBC at pH 2.0 demonstrated strong positive correlations with ergosterol and L-carnitine (r ≥ 0.85). WHC was highly positively correlated with ergosterol (r = 0.96), and CBC at pH 7.0 was positively correlated with L-carnitine. Regarding NO inhibition activity in LPS-stimulated RAW264.7 macrophages, there was a strong positive correlation with L-carnitine (r = 0.86), β-sitosterol (r = 0.90), WHC (r = 0.8), WSC (r = 0.88), OAC (r = 0.88), CBC at pH 2.0 (r = 0.89), and CBC at pH 7.0 (r = 0.98) [Fig. 6(A)]. Furthermore, Principal component analysis (PCA), an unsupervised multivariate technique that transforms correlated variables into a few orthogonal principal components representing the primary sources of variation, was additionally conducted to assess the overall connections among fermentation time, phytochemical content, and biological activities [Fig. 6(B)]. In Fig. 6(B), PC1 and PC2 accounted for 60.90% and 31.38% of the total variance, respectively, explaining 92.28% of the cumulative variance. Variables located on the negative side of PC1 were characterized by high negative loadings for TFC, FRAP, and chlorogenic acid, whereas those on the positive side of PC1 were characterized by positive loadings for ergosterol, L-carnitine, β-sitosterol, WHC, WSC, OAC, CBC at pH 2.0 and 7.0, and NO inhibition. In addition, ABTS and quinic acid exhibited negative loadings on PC2, whereas TPC, TAA, ORAC, WHC, and caffeic acid exhibited positive loadings on PC2 [Fig. 6(B)]. The PCA results revealed distinct metabolic shifts across the fermentation stages, reflecting the biochemical changes in *E. senticosus* leaves during fermentation by *R. oligosporus*. The temporal progression of the samples in the PCA score plot was associated with changes in phytochemical composition, antioxidant capacity, and anti-inflammatory activity, highlighting the influence of fermentation time on the release, biotransformation, and accumulation of bioactive compounds. Notably, samples from the later fermentation stages were positively associated with L-carnitine, β-sitosterol, and increased inhibition of nitric oxide (NO) production, suggesting that fungal metabolism promotes the accumulation of metabolites associated with enhanced biological activity. Overall, these findings indicate that *R. oligosporus*-mediated metabolic remodeling improves the functional properties of fermented *E. senticosus* leaves and supports solid-state fermentation as an effective strategy for valorizing this underutilized biomass. In summary, solid-state fermentation (SSF) of *E. senticosus* leaves using *R. oligosporus* improved their physicochemical properties and enhanced their functional potential. This bioprocess promoted the accumulation of important bioactive compounds, including L-carnitine (15.67 mg/kg DM), ergosterol (107.92 mg/kg DM), caffeic acid (4356.84 mg/kg DM), and β-sitosterol (5503.91 mg/kg DM), while increasing antioxidant activity (8.8–30.4%) and suppressing nitric oxide (NO) production in LPS-stimulated RAW264.7 macrophages. Collectively, these findings suggest that *R. oligosporus*-mediated fermentation effectively valorizes underutilized *E. senticosus* leaf biomass by enhancing its functional properties, thereby supporting its potential application as a functional food ingredient. As a future perspective, pilot-scale studies are needed to validate the production efficiency, cost-effectiveness, and process consistency of the optimized 72-h SSF process. Although *R. oligosporus* is Generally Recognized as Safe (GRAS), comprehensive toxicological evaluation of the fermented *E. senticosus* leaf product will be required before commercial application. Based on the present findings, a 72-h fermentation appears to provide the best balance between enhanced antioxidant activity and anti-inflammatory efficacy, making it a promising processing condition for the industrial production of functional *E. senticosus* ingredients.

## Supplementary Information

Below is the link to the electronic supplementary material.

Supplementary file1 (DOCX 528 kb)

## References

[CR1] Adhimoolam K, Sureshbabu A, Smirnova E, Muthuramalingam P, Thi CTD, Senthil K, Min T. β-Sitosterol-Dietary sources and role in cancer and diabetes management. Food Science & Nutrition. 12: 8870-8886 (2024) 10.1002/fsn3.4380 PMC11606809 39619995

[CR2] Arancibia-Diaz A, Astudillo-Castro C, Altamirano C, Vergara-Castro M, Soto-Maldonado C, Cordova A, Fuentes P, Zuniga-Hansen ME, Bravo J. Enhanced antioxidant capacity and yield of release of chlorogenic acids and derivates by solid-state fermentation of spent coffee ground under controlled conditions of aeration and moisturizing. Food Chemistry. 479: 143744 (2025) 10.1016/j.foodchem.2025.143744 40068539

[CR101] Arslan-Tontul S, Yang S, Casertano M, Pellegrini N, Fogliano V. Upcycling of spent coffee grounds solid-state fermentation by Aspergillus strains: In vitro assessment of prebiotic activity and gut health benefits. Food Chemistry: 147310 (2025). 10.1016/j.foodchem.2025.147310 41344235

[CR3] Chandimali N, Bak SG, Park EH, Lim HJ, Won YS, Kim EK, Park SI, Lee SJ. Free radicals and their impact on health and antioxidant defenses: A review. Cell Death Discovery. 11: 19 (2025) 10.1038/s41420-024-02278-8 PMC11760946 39856066

[CR4] Chang CC, Yang MH, Wen HM, Chern JC. Estimation of total flavonoid content in propolis by two complementary colorimetric methods. Journal of Food and Drug Analysis. 10: 178-182 (2002)

[CR5] Chilakamarry CR, Sakinah AM, Zularisam A, Sirohi R, Khilji IA, Ahmad N, Pandey A. Advances in solid-state fermentation for bioconversion of agricultural wastes to value-added products: Opportunities and challenges. Bioresource Technology. 343: 126065 (2022) 10.1016/j.biortech.2021.126065 34624472

[CR6] Correia RTP, McCue P, Magalhaes MMA, Macedo GR, Shetty K. Production of phenolic antioxidants by the solid-state bioconversion of pineapple waste mixed with soy flour using *Rhizopus oligosporus*. Process Biochemistry. 39: 2167-2172 (2004)

[CR7] Dulf FV, Vodnar DC, Dulf EH, Tosa MI. Total phenolic contents, antioxidant activities, and lipid fractions from berry pomaces obtained by solid-state fermentation of two sambucus species with *Aspergillus niger*. Journal of Agricutlural and Food Chemistry. 63: 3489-500 (2015) 10.1021/acs.jafc.5b00520 25787023

[CR8] Dulf FV, Vodnar DC, Socaciu C. Effects of solid-state fermentation with two filamentous fungi on the total phenolic contents, flavonoids, antioxidant activities and lipid fractions of plum fruit (*Prunus domestica L*.) by-products. Food Chemistry. 209: 27–36 (2016) 10.1016/j.foodchem.2016.04.016 27173530

[CR100] Egbune EO, Avwioroko OJ, Orororo OC, Egbune OU, Aganbi E, Anigboro AA, Tonukari NJ. Rhizopus oligosporus alkaline protease in cassava fermentation: Characterization and detergent potential. Biocatalysis and Agricultural Biotechnology 54: 102954 (2023). 10.1016/j.bcab.2023.102954

[CR102] Elantary R, Othman S. Role of L-carnitine in cardiovascular health: literature review. Cureus 16 (2024). 10.7759/cureus.70279 PMC11427024 39329040

[CR9] Gulcin I. Antioxidant and antiradical activities of L-carnitine. Life Science. 78: 803-11 (2006) 10.1016/j.lfs.2005.05.103 16253281

[CR10] Hur J, Nguyen TTH, Park N, Kim J, Kim D. Characterization of quinoa (*Chenopodium quinoa*) fermented by *Rhizopus oligosporus* and its bioactive properties. AMB Express. 8: 143 (2018) 10.1186/s13568-018-0675-3 PMC6134574 30203194

[CR11] Jia MY, Chen JJ, Liu XZ, Xie MY, Nie SP, Chen Y, Xie JH, Yu Q. Structural characteristics and functional properties of soluble dietary fiber from defatted rice bran obtained through *Trichoderma viride* fermentation. Food Hydrocolloids. 94: 468-474 (2019)

[CR12] Kim H, Hur S, Lim J, Jin K, Yang TH, Keehm IS, Kim SW, Kim T, Kim D. Enhancement of the phenolic compounds and antioxidant activities of Kombucha prepared using specific bacterial and yeast. Food Bioscience. 56: 103431 (2023)

[CR13] Kim H, Jeon J, Lee J, Song C, Gu B, Kim NM, Yang TH, Oh S, Park S, Pal K, Kim GJ, Kim D. Utilizing kombucha culture for coffee fermentation and biochemical characteristic analysis. Current Research in Food Science. 10: 100996 (2025a) 10.1016/j.crfs.2025.100996 PMC11867273 40018374

[CR14] Kim H, Jin K, Lee JY, Yang TH, Oh S, Park S, Tran GB, Le HT, Pal K, Kim GJ, Kim D. Functional properties of hard kombucha brewed with deep ocean water using *Lachancea thermotolerans*. Scientific Reports. 15: 26959 (2025b) 10.1038/s41598-025-12171-8 PMC12290105 40707548

[CR15] Kim H, Kang CG, Seong H, Kim T, Jo J, Kim GHJ, Kim SW, Kim D. Enhancement of the water solubility of curcuminoids by micelle formulations with ginsenosides to improve antioxidant activity, suppress nitric oxide release, and reduce lipid accumulation. Journal of Functional Foods. 116: 106186 (2024)

[CR16] Krishna C. Solid-state fermentation systems—an overview. Critical Reviews in Biotechnology. 25: 1-30 (2005) 10.1080/07388550590925383 15999850

[CR17] Kwak SH, Kim H, Jeon JH, Pal K, Kang DH, Kim D. Phytochemical and functional characterization of fermented Yerba mate using *Rhizopus oligosporus*. AMB Express. 13: 94 (2023) 10.1186/s13568-023-01600-4 PMC10492770 37689820

[CR18] Kwon RH, Na H, Kim JH, Kim SA, Kim SY, Jung HA, Lee SH, Wee CD, Lee KS, Kim HW. Comprehensive profiling of phenolic compounds and triterpenoid saponins from *Acanthopanax senticosus* and their antioxidant, α-glucosidase inhibitory activities. Scientific Reports. 14: 26330 (2024) 10.1038/s41598-024-77574-5 PMC11530669 39487169

[CR19] Lee G, Nguyen TTH, Lim TY, Lim J, Park B, Lee S, Mok IK, Pal K, Lim S, Kim D. Fermented wild ginseng by *Rhizopus oligosporus* improved L-carnitine and ginsenoside contents. Molecules. 25: 2111 (2020) 10.3390/molecules25092111 PMC7249200 32365963

[CR20] Lee JY, Kim H, Yang TH, Oh S, Kim GJ, Park S, Lee S, Mok IK, Kang DH, Kim D. Enhancing the biochemical and functional properties of water kefir: Comparative effects of different water sources with emphasis on deep ocean water. Journal of Functional Foods. 132: 106965 (2025)

[CR21] Lee LW, Cheong MW, Curran P, Yu B, Liu SQ. Modulation of coffee aroma via the fermentation of green coffee beans with *Rhizopus oligosporus*: II. Effects of different roast levels. Food Chemistry. 211: 925–36 (2016) 10.1016/j.foodchem.2016.05.073 27283714

[CR22] Lee S, Kim BK, Cho SH, Shin KH. Phytochemical constituents from the fruits of *Acanthopanax sessiliflorus*. Archives of Pharmacal Research. 25: 280-284 (2002) 10.1007/BF02976626 12135097

[CR23] Leonard W, Zhang P, Ying D, Adhikari B, Fang Z. Fermentation transforms the phenolic profiles and bioactivities of plant-based foods. Biotechnology Advances. 49: 107763 (2021) 10.1016/j.biotechadv.2021.107763 33961978

[CR24] Lim J, Kim H, Park SB, Pal K, Kim SW, Kim D. Effects of solid-state fermentation using *R. oligosporus* on the phytochemical composition of wild-simulated ginseng leaf and its biological properties. Food Bioscience. 52: 102412 (2023)

[CR25] Lim J, Nguyen TTH, Pal K, Gil Kang C, Park C, Kim SW, Kim D. Phytochemical properties and functional characteristics of wild turmeric (*Curcuma aromatica)* fermented with *Rhizopus oligosporus*. Food Chemistry X. 13: 100198 (2022) 10.1016/j.fochx.2021.100198 PMC9039939 35499023

[CR26] Lin Y, Liu L, Li L, Xu Y, Zhang Y, Zeng H. Properties and digestibility of a novel porous starch from lotus seed prepared via synergistic enzymatic treatment. International Journal of Biological Macromolecules. 194: 144-152 (2022) 10.1016/j.ijbiomac.2021.11.196 34863826

[CR27] Liu S, Wang X, Tang X, Fang W. Histone deacetylase HDAC3 regulates ergosterol production for oxidative stress tolerance in the entomopathogenic and endophytic fungus *Metarhizium robertsii*. Msystems. 9:e00953-24 (2024) 10.1128/msystems.00953-24 PMC11494875 39287372

[CR28] Liu Y, Wang Z, Wang C, Si H, Yu H, Li L, Fu S, Tan L, Li P, Liu J. Comprehensive phytochemical analysis and sedative-hypnotic activity of two *Acanthopanax* species leaves. Food & Function. 12: 2292-2311 (2021) 10.1039/d0fo02814b 33605281

[CR29] Ma W, Liang Y, Lin H, Chen Y, Xie J, Ai F, Yan Z, Hu X, Yu Q. Fermentation of grapefruit peel by an efficient cellulose-degrading strain, (*Penicillium YZ-1*): Modification, structure and functional properties of soluble dietary fiber. Food Chemistry. 420: 136123 (2023) 10.1016/j.foodchem.2023.136123 37094537

[CR30] Nicol A, Kwok RTK, Chen C, Zhao W, Chen M, Qu J, Tang BZ. Ultrafast delivery of aggregation-induced emission nanoparticles and pure organic phosphorescent nanocrystals by saponin encapsulation. Journal of the American Chemical Society. 139: 14792-14799 (2017) 10.1021/jacs.7b08710 28960975

[CR31] Niu J, Alazi E, Reid ID, Arentshorst M, Punt PJ, Visser J, Tsang A, Ram AF. An evolutionarily conserved transcriptional activator-repressor module controls expression of genes for d-galacturonic acid utilization in *Aspergillus niger*. Genetics. 205: 169-183 (2017) 10.1534/genetics.116.194050 PMC5223501 28049705

[CR32] Ou B, Hampsch-Woodill M, Prior RL. Development and validation of an improved oxygen radical absorbance capacity assay using fluorescein as the fluorescent probe. Journal of Agricultural and Food Chemistry. 49: 4619-4626 (2001) 10.1021/jf010586o 11599998

[CR33] Pandey A. Solid-state fermentation. Biochemical Engineering Journal. 13: 81-84 (2003) 10.1016/s1369-703x(00)00065-6 10908866

[CR34] Pandey A, Soccol CR, Mitchell D. New developments in solid state fermentation: I-bioprocesses and products. Process Biochemistry. 35: 1153-1169 (2000)

[CR35] Pekala J, Patkowska-Sokola B, Bodkowski R, Jamroz D, Nowakowski P, Lochynski S, Librowski T. L-carnitine-metabolic functions and meaning in human life. Current Drug Metabolism. 12: 667-678 (2011) 10.2174/138920011796504536 21561431

[CR36] Re R, Pellegrini N, Proteggente A, Pannala A, Yang M, Rice-Evans C. Antioxidant activity applying an improved ABTS radical cation decolorization assay. Free Radical Biology and Medicine. 26: 1231-1237 (1999) 10.1016/s0891-5849(98)00315-3 10381194

[CR37] Reuter SE, Evans AM. Carnitine and acylcarnitines: pharmacokinetic, pharmacological and clinical aspects. Clinical Pharmacokinetics. 51: 553-572 (2012) 10.1007/BF03261931 22804748

[CR38] Ryu KM, Kim H, Woo J, Lim J, Kang CG, Kim SW, Kim T, Kim D. Enhancement of the bioactive compounds and biological activities of maca (*Lepidium meyenii*) via solid-state fermentation with *Rhizopus oligosporus*. Food Science and Technology. 33: 2585-2596 (2024) 10.1007/s10068-023-01508-6 PMC11319679 39144202

[CR39] Saeed N, Khan MR, Shabbir M. Antioxidant activity, total phenolic and total flavonoid contents of whole plant extracts *Torilis leptophylla L*. BMC Complementary and Alternative Medicine. 12: 221 (2012) 10.1186/1472-6882-12-221 PMC3524761 23153304

[CR40] Sharmin F, Koyama T, Koyama H, Ishizaki S. Cholesterol-binding ability of saponin from Japanese starfish. Journal of Food Science and Technology. 58: 3056-3064 (2021) 10.1007/s13197-020-04809-4 PMC8249638 34294968

[CR41] Shi C, Liang Z, Li T, Hao Q, Xiang H, Xie Q. Metabolome and microbiome analyses of the anti-fatigue mechanism of *Acanthopanax senticosus* leaves. Food Function. 15: 3791-3809 (2024) 10.1039/d3fo05311c 38511300

[CR42] Sivaramakrishnan R, Ramprakash B, Ramadoss G, Suresh S, Pugazhendhi A, Incharoensakdi A. High potential of *Rhizopus* treated rice bran waste for the nutrient-free anaerobic fermentative biohydrogen production. Bioresource Technology. 319: 124193 (2021) 10.1016/j.biortech.2020.124193 33035864

[CR43] Steudler S, Böhmer U, Weber J, Bley T. Biomass measurement by flow cytometry during solid‐state fermentation of *Basidiomycetes*. Cytometry Part A. 87: 176-188 (2015) 10.1002/cyto.a.22592 25475642

[CR44] Sun CC, Wu XF, Chen XJ, Li XJ, Zheng Z, Jiang SW. Production and characterization of okara dietary fiber produced by fermentation with *Monascus anka*. Food Chemistry. 316: 126243 (2020) 10.1016/j.foodchem.2020.126243 32036177

[CR45] Swiegers JH, Vaz FM, Pretorius IS, Wanders RJ, Bauer FF. Carnitine biosynthesis in *Neurospora crassa*: identification of a cDNA coding for epsilon-N-trimethyllysine hydroxylase and its functional expression in *Saccharomyces cerevisiae*. FEMS Microbiology Letters. 210: 19-23 (2002) 10.1111/j.1574-6968.2002.tb11154.x 12023072

[CR46] Tosh SM, Yada S. Dietary fibers in pulse seeds and fractions: Characterization, functional attributes, and applications. Food Research International. 43: 450-460 (2010)

[CR47] Varzakas T. *Rhizopus oligosporus* mycelial penetration and enzyme diffusion in soya bean tempe. Process Biochemistry. 33: 741-747 (1998)

[CR103] Virmani MA, Cirulli M. The role of L-carnitine in mitochondria, prevention of metabolic inflexibility and diseaseinitiation. International Journal of Molecular Sciences 23: 2717 (2022). 10.3390/ijms23052717 PMC8910660 35269860

[CR48] Zhang Y, Wei R, Azi F, Jiao L, Wang H, He T, Liu X, Wang R, Lu B. Solid-state fermentation with *Rhizopus oligosporus* RT-3 enhanced the nutritional properties of soybeans. Frontiers Nutrition. 9: 972860 (2022) 10.3389/fnut.2022.972860 PMC9493129 36159501

[CR49] Zhang YZ, Yu K, Fang GY, Lu BY, Azi F, Gao ZD, Zhou YX, Wu FH, Hu H, Sun XT, Liu XQ. Improvement of the nutritional composition, flavor characteristic, and functional activity of brown rice bio-processed with *R. oligosporus*. Journal of Cereal Science. 129: 104435 (2026)

