# Supplementary material for: Transformation of phytochemicals and functional properties in Eleutherococcus senticosus leaves during solid-state fermentation with Rhizopus oligosporus
Source: Food Sci Biotechnol. 2026 Aug 13;35(13):3719–34. doi: 10.1007/s10068-026-02276-9 (PMC13731549; doi:10.1007/s10068-026-02276-9)
Supplement: Supplementary file 1 [file 10068_2026_2276_MOESM1_ESM.docx]

**Supplementary materials**

**Transformation of phytochemicals and functional properties in *Eleutherococcus senticosus* leaves during solid-state fermentation with *Rhizopus oligosporus***

**Suprakash De ^1^, Hayeong Kim^2*^, Ghahyun Jeffrey Kim^3^, Taeyoung Um^4^, Yedam Moon^2^, Kibeom Jin^2^, Sooye An^5^, Kunal Pal^6,^, Doman Kim^1, 2, 7,*^**

^1^Graduate School of International Agricultural Technology, Seoul National University, Gangwon-do, 25354, Republic of Korea

^2^Institutes of Green Bioscience and Technology, Seoul National University, Gangwon-do, 25354, Republic of Korea

^3^Department of Clinical Pharmacy Practice, School of Pharmacy &Pharmaceutical Sciences, UC Irvine, CA, 92617, USA

^4^Department of Plant Science, Kangwon National University, Gangneung, 25457, Republic of Korea

5Agricultural corporation Soye Pyeongchang Ogapi Co. Ltd, Pyeongchang, 25354, Republic of Korea

^6^Falculty Department of Biotechnology and Medical Engineering, National Institute of Technology Rourkela, Rourkela 769008, Odisha, India

^77^ Fervere Campus Corporation, Gangwon-do, 25354, Republic of Korea

**Corresponding Author:**

**Hayeong Kim:** E-mail: hara2910@snu.ac.kr **Tel:** +82-33-339-5736; **Fax:** +82-33-339-5716

**Doman Kim:** **E-mail**: [kimdm@snu.ac.kr](mailto:kimdm@snu.ac.kr); **Tel:** +82-33-339-5720; **Fax:** +82-33-339-5716

Suprakash De: suprakash@snu.ac.kr

Hayeong Kim: hara2910@snu.ac.kr

Ghahyun Jeffrey Kim: jeffrey.kim@uci.edu

Taeyoung Um: phytosonic@kangwon.ac.kr

Yedam Moon:	ansdpekfa@snu.ac.kr

Kibeom Jin: usb95@snu.ac.kr

Sooye An: goodogapi@naver.com

Kunal Pal: kpal.nitrkl@gmail.com

Doman Kim: kimdm@snu.ac.kr

**A running title: Fermentation-induced changes in *E. senticosus* leaves**

**Table S1.** Calibration curves, linearity (R²), limit of detection (LOD), and limit of quantification (LOQ) for L-carnitine analysis by UPLC-MS

| Calibration curve | Calibration equation | R^2^ |
| --- | --- | --- |
| 1 | Y = 2.03 × 10^7^X + 1.54 × 10^6^ | 0.99 |
| 2 | Y = 1.99 × 10^7^X + 1.55 × 10^6^ | 0.99 |
| 3 | Y = 2.03 × 10^7^X + 1.59 × 10^6^ | 0.99 |
| LOD (ng/mL ) | | 4.3 |
| LOQ (ng/mL ) | | 13 |

**Fig. S1. Chromatogram analysis of Ergosterol standard (A) and fermented *E. senticosus* leaf at 0 h (B) and 120 h (C) using UPLC-MS with a BEH C_18_ column, using isocratic elution with acetonitrile as the mobile phase at a flow rate of 0.1 mL/min for 11 min.**

**Fig. S2. Chromatogram analysis of L-carnitine standard (A) and fermented *E. senticosus* leaf at 0 h (B) and 120 h (C) using UPLC-MS with a BEH HILIC column, using acetonitrile with 0.1% (v/v) formic acid (A) and 15 mM ammonium formate with 0.1% (v/v) formic acid (B) as the mobile phase.**

**Fig. S3. Mechanism for changes in various bioactive compounds.**
